# Gga-miR-130b-3p inhibits MSB1 cell proliferation, migration, invasion, and its downregulation in MD tumor is attributed to hypermethylation

**DOI:** 10.18632/oncotarget.24679

**Published:** 2018-05-11

**Authors:** Chunfang Zhao, Xin Li, Bo Han, Lujiang Qu, Changjun Liu, Jiuzhou Song, Ling Lian, Ning Yang

**Affiliations:** ^1^ Department of Animal Genetics and Breeding, College of Animal Science and Technology, China Agricultural University, Beijing 100193, China; ^2^ College of Animal Science and Veterinary Medicine, Tianjin Agricultural University, Tianjin 300384, China; ^3^ Division of Avian Infectious Diseases, Harbin Veterinary Research Institute of Chinese Academy of Agricultural Sciences, Harbin 150001, China; ^4^ Department of Animal & Avian Sciences, University of Maryland, College Park, Maryland 20742, United States

**Keywords:** gga-miR-130b-3p, methylation, proliferation, migration, invasion

## Abstract

Marek's disease is an oncogenic and lymphoproliferative disease of chickens caused by Marek's disease virus. Hypermethylation or hypomethylation of CpG islands in gene promoter region are involved in the initiation and progression of carcinogenesis. In this study, we analyzed differential methylation levels of upstream region of gga-miR-130b-3p gene between Marek's disease virus-infected tumorous and non-infected spleens. Around the upstream 1 kb of gga-miR-130b-3p gene, two amplicons were designed that covered 616 bp. There were forty-eight CpG sites in this region. CpG sites in this region presented higher methylation level in tumorous spleens compared with that in non-infected ones. There were eight CpG sites significantly hypermethylated in tumorous spleens. The expression level of three DNA methyltransferases including DNMT1, DNMT3a and DNMT3b increased and the expression level of Tet ten-eleven translocation protein 2 remarkably decreased in tumorous spleens. Hypermethylation in the upstream region of gga-miR-130b-3p gene might be a direct reason for its downregulation in MD tumorous tissues. Moreover, cell proliferation of Marek's disease lymphoblastoid cell line MDCC-MSB1 was remarkably inhibited at 24, 36, 48, 60 and 72 h post-gga-miR-130b-3p-agomir transfection. The transwell migration assay indicated cell number of migration was significantly lower in miRNA agomir transfection group. Matrix metalloproteinases MMP2 and MMP9 are involved in tumor invasion, and their protein levels were significantly downregulated at 72 h post-miRNA-agomir transfection. Collectively, these results indicated that hypermethylation in upstream region of gga-miR-130b-3p gene contributed to its downregulation in tumorous tissues. Gga-miR-130b-3p plays an inhibitory role in lymphomatous cell transformation.

## INTRODUCTION

Marek's disease (MD) in chickens is a critical disease that threatens the development of poultry, which is caused by oncogenic herpes virus Marek's disease virus (MDV). This disease results in various clinical syndromes and is mainly characterized by immunosuppression, neuritis, and rapid-onset neoplastic T-cell lymphomas formation in multiple visceral organs [1–5]. Natural infection with MDV *in vivo* was typically divided into four phases, including the cytolytic infection stage (3 to 6 days post-infection [d.p.i.]), the latent infection stage (7 to 10 d.p.i.), the further cytolytic infection stage (10 to 14 d.p.i.) and the tumor transformation stage (after 21 d.p.i.) [1, 2].

MicroRNA (miRNA) is a class of small non-coding single-stranded RNA (approximately 22 nucleotides in length), which exerts post-transcriptional regulation of target gene expression by interacting with the 3′-untranslated region of mRNA and suppressing mRNA translation, or a combination of both mechanisms [6–8]. Interaction between miRNA and target genes of miRNA at the post-transcriptional level provides fine-tuned dynamic regulation of cell signalling pathways [9]. miRNA is involved in various biological processes, including differentiation, development and tumorigenesis [10]. DNA methylation is a type of epigenetic modification that entails the covalent addition of a methyl group to the cytosine residue within the CpG context through the catalysis of DNA methyltransferases, which affects gene expression without genetic alterations [11]. Both miRNA and DNA methylation are crucial regulators of gene expression. The miRNA expression dysregulation in some cancers is an outcome of aberrant methylation states in the promotor regions of the miRNAs [11–14]. In fact, miRNAs act as both the effectors and targets in the DNA methyltransferases (*DNMTs*). MiRNAs can target *DNMT* mRNA and inhibit their expression, which results in variations on genome-wide methylation patterns [11, 15, 16].

Nowadays, there are numerous reports about the methylation in the promoter of miRNA in malignant tumors in mammals. Although both host and viral miRNAs are specifically implicated in MD tumorigenesis, similar study is limited and most studies were focused on global host and viral gene methylation [17–20]. The study of DNA methylation in MD has been carried out for a long time. Gathered data shows that DNA methylation have an impact on maintaining viral latency of MDV in the propagation process *in vivo* and in MDV-transformed lymphoblastoid cell lines *in vitro* [21, 22]. Tian et al. (2013) reported that DNA methylation levels *in vitro* are associated with MDV replication, and MDV propagation in the infected DF-1 cells is restricted by methylation inhibitor 5-azacytidine [23]. *CD4* gene expression level was directly related to T cell development and its methylation level in the promoter was downregulated in MD susceptible chickens [24]. MDV infection induced dynamic promotor methylation alterations and methylation variations were observed in the MD progression. By bisulfite pyrosequencing method, a few candidate genes were found to be involved in cell adhesion, immune system process, and responding to stimulus and higher methylated in the MD-susceptible 7_2_ line than in the MD-resistant 6_3_ line [25]. Hodgkin's disease antigen *CD30* is over-expressed in chickens with MD and its overexpression results from hypomethylation in the promotor [26, 27].

MiR-130b functions as an oncogenic or tumor suppressor miRNA and shows dysregulation in various cancers including gastric, endometrial, and lung cancer [28–30]. Our previous study found gga-miR-130b-3p was abnormally expressed in MD tumorous tissues. To elucidate the reason for its differential expression and its function in MD tumorigenesis, we analyzed the methylation pattern of gga-miR-130b-3p gene and detected the effect of gga-miR-130b-3p on main features of MD lymphoblastoid cell line MDCC-MSB1.

## RESULTS

### The upstream region of gga-miR-130b-3p gene was hypermethylated in MDV-infected tumorous spleens

**Figure 1:**
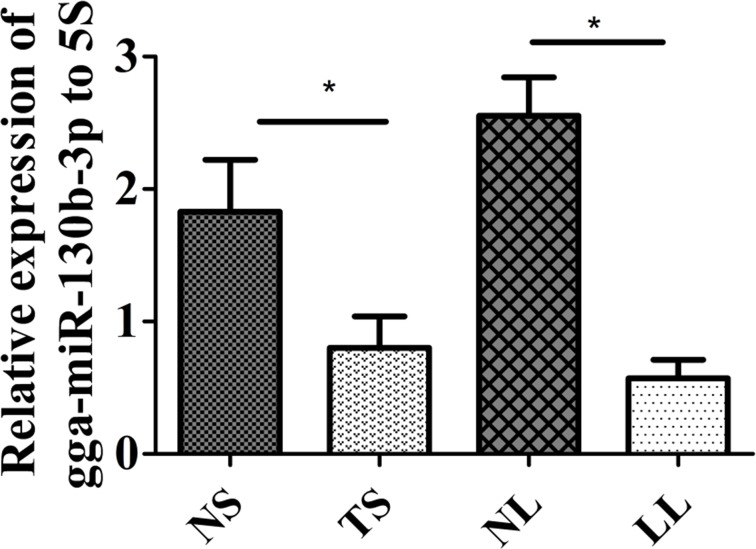
Expression level of gga-miR-130b-3p in MDV-infected tumorous and non-infected tissues Expression level of gga-miR-130b-3p in non-infected spleen (NS), tumorous spleen (TS), non-infected liver (NL) and MD lymphoma from liver (LL) (n = 8). The data are expressed as the mean± S.E. ^*^P<0.05.

**Figure 2:**
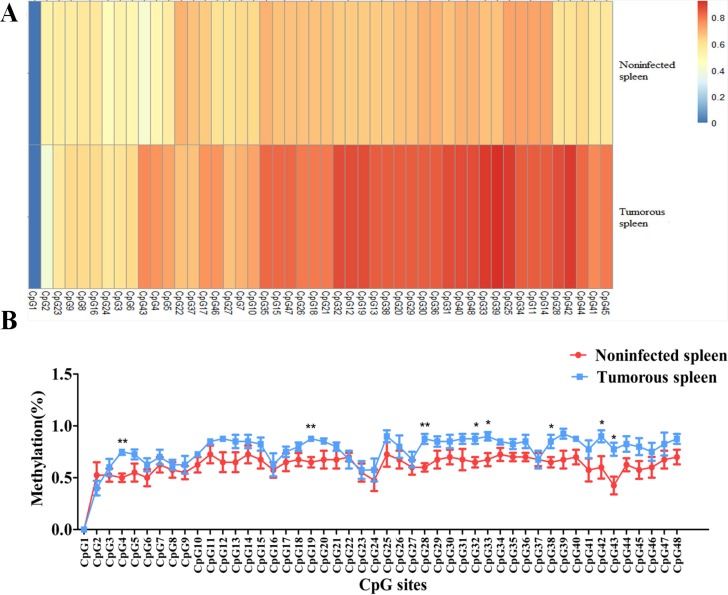
Methylation level of each CpG site in the upstream region of gga-miR-130b-3p gene between MDV-infected tumorous and non-infected spleens **(A)** Heat map depicting methylation pattern of forty-eight CpG sites in the upstream region of gga-miR-130b-3p gene between tumorous and non-infected spleens. **(B)** Line chart depicting methylation pattern of forty-eight CpG sites in the upstream region of gga-miR-130b-3p gene between tumorous and non-infected spleens. The data are expressed as the mean± S.E. ^*^P<0.05. ^**^P<0.01.

Our previous study investigated miRNA expression profiling between MD lymphoma and non-infected samples of chickens using Solexa deep sequencing, which showed that gga-miR-130b-3p expression was downregulated in MD lymphoma samples compared with that in non-infected ones. Its expression level was further determined between tumorous tissues and non-infected ones using qRT-PCR. The expression level of gga-miR-130b-3p was significantly downregulated in tumorous spleens and livers compared with that in non-infected samples, respectively (Figure 1). The methylation pattern for the upstream 1 kb region of gga-miR-130b-3p gene was analyzed. Two amplicons covering 616 bp around this region were designed. There were forty-eight CpG sites in the upstream region of gga-miR-130b-3p gene. The methylation difference of each CpG site between tumorous and non-infected spleens was further compared. Though the methylation levels varied at different CpG sites, overall methylation level in tumorous spleens was higher than that in non-infected ones (Figure 2A). It was not methylated at CpG site 1 at both tumorous and non-infected spleens. The rate of methylation was significantly higher at CpG site 4, 19, 28, 32, 33, 38, 42 and 43 in tumorous spleens compared with that in non-infected ones. CpG site 39 showed the highest methylation level (92.5%), whereas CpG site 2 had the lowest methylation level (40%) (Figure 2B).We identified the transcription factor binding sites using MatInspector (Supplementary Table 1) [31]. Among them, some transcription factor binding sites existed in immune system and were involved in immune response, cell cycle regulation and tumorigenesis. The transcriptional factor stimulating protein 1 (*SP1*) binding site was predicted in the amplicon. There were four CpG sites in the *SP1* binding site. The *SP1* binding site was located at the CpG site 24, 25, 26, 27 in the upstream region of gga-miR-130b-3p gene and their methylation rate was 0.575, 0.900, 0.800, 0.675, respectively in tumorous spleens.

### DNMTs expression level was upregulated and TETs expression level was downregulated in MDV-infected tumorous spleens

**Figure 3:**
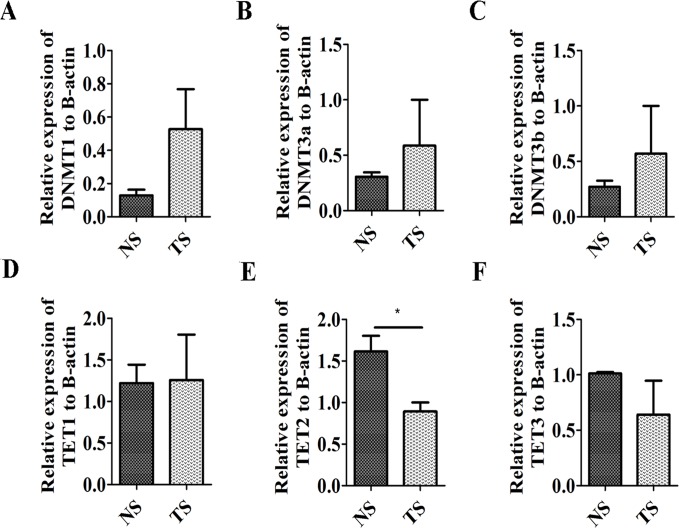
Expression level of DNA methyltransferases and Tet ten-eleven translocation proteins **(A, B, C)** Expression level of *DNMT1* (A), *DNMT3a* (B) and *DNMT3b* (C) in non-infected spleens (NS) and tumorous spleens (TS). **(D, E, F)** Expression level of *TET1* (D), *TET2* (E) and *TET3* (F) in non- infected spleens (NS) and tumorous spleens (TS). The data are expressed as the mean± S.E. ^*^P<0.05.

To reinforce the results above, the expression level of *DNMTs* and enzymes involved in demethylation was further analyzed. *DNMTs* expression levels including *DNMT1*, *DNMT3a* and *DNMT3b* were upregulated in MDV-infected tumorous spleens compared with that in non-infected ones (Figure 3A–3C). The enzymes Tet ten-eleven translocation (*TET*) proteins involved in demethylation include *TET1*, *TET2* and *TET3*, and *TET2* expression was significantly downregulated in tumorous spleens while *TET1* and *TET3* expression had no significant differences between tumorous and non-infected spleens (Figure 3D–3F).

### Gga-miR-130b-3p inhibited MSB1 cell proliferation, migration and invasion

**Figure 4:**
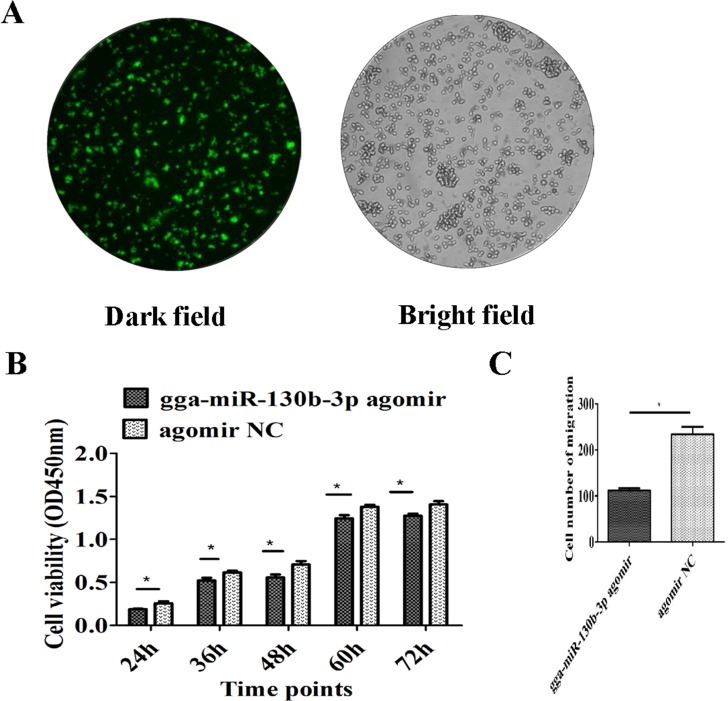
Effect of gga-miR-130b-3p on cell proliferation and migration **(A)** Depiction of the miRNA agomir or agomir NC transfection efficiency. To show transfection efficiency of the agomir or agomir NC, a transfection reagent was used to transfect MSB1 cells with FAM-labelled agomir NC. A fluorescence microscope was used to observe the cells, which were illuminated under a bright or dark field with magnification at 100×. **(B)** Effect of gga-miR-130b-3p on MSB1 cell proliferation. Cell proliferation was detected by a CCK-8 assay at 24, 36, 48, 60 and 72 h after transfection with the gga-miR-130b-3p agomir or NC (n = 5). **(C)** Effect of gga-miR-130b-3p on MSB1 cell migration. Transwell migration assay performed after transduction of the gga-miR-130b-3p agomir or NC (n = 2). The data are expressed as the mean± S.E. ^*^P<0.05.

**Figure 5:**
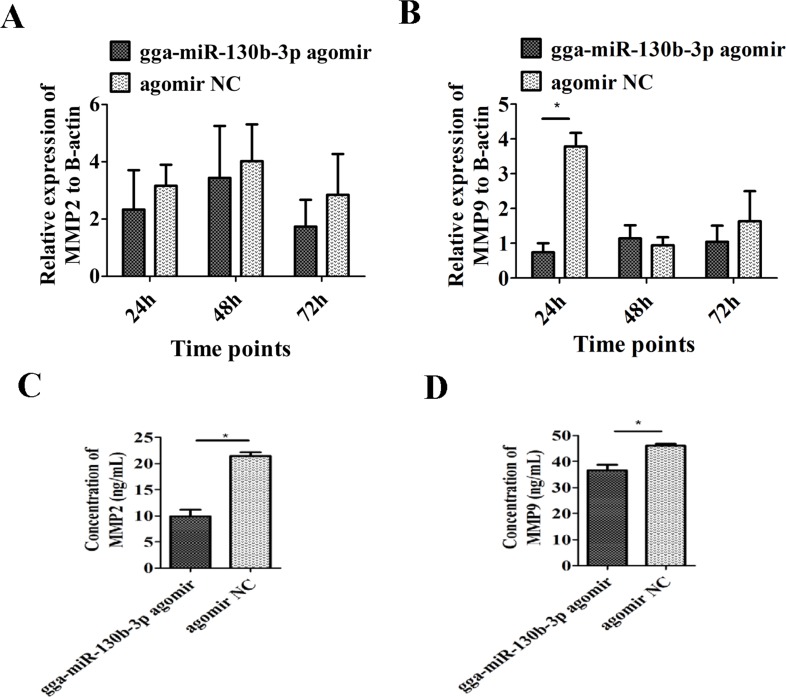
Effect of gga-miR-130b-3p on cell invasion mRNA level of MMP2 **(A)** and MMP9 **(B)** after transduction of the gga-miR-130b-3p agomir or NC (n = 3). The protein level of MMP2 **(C)** and MMP9 **(D)** after transduction of the gga-miR-130b-3p agomir or NC (n = 3).The data are expressed as the mean± S.E. ^*^P<0.05. ^**^P<0.01.

The MSB1 cells were transfected with the gga-miR-130b-3p agomir to stimulate miRNA overexpression. The efficiency of transfection reached up to 70% (Figure 4A). Cell proliferation was lower in cultures at 24, 36, 48, 60 and 72 h post-agomir transfection than that in the NC transfection group (Figure 4B). The migration cell number significantly decreased when MSB1 cells were transfected with agomir (Figure 4C). The expression levels of two genes, *MMP2* and *MMP9*, that are closely correlated to cell invasion were examined by qRT-PCR and ELISA. mRNA expression of *MMP2* had no significant differences post-agomir transfection (Figure 5A). mRNA expression of *MMP9* was significantly downregulated at 24 h and had no significant differences at 48 and 72 h post-agomir transfection (Figure 5B). The protein levels of both MMP2 and MMP9 significantly decreased post-agomir transfection at 72 h (Figure 5C, 5D).

## DISCUSSION

Host and viral miRNAs as well as DNA methylation involved in Marek's disease tumorigenesis have been broadly reported. The study about DNA methylation was mostly focused on methylation status of viral and host coding genes. Latent MDV1 DNA is considerably methylated in MDV-transformed cell lines, which contributes to the repression of viral gene transcription [22, 32]. However, MDV lytic genes like *pp34* and *pp28* is mostly non-methylated in MSB1 cells [33]. Yu et al. (2008) firstly analyzed the DNA methylation profiles of *DNMTs* in chickens with different MD resistance and provided tissue-specific methylation patterns of *DNMT3a* and age-specific methylation of *DNMT1* [34]. Tian et al. (2013) mapped the chicken genome-wide DNA methylation profiles by methylation mapping analysis by paired-end sequencing and provides a more comprehensive picture of the chicken methylome [23]. Li et al. (2015) established a single-base resolution DNA methylation profile of chicken lung using whole-genome bisulfite sequencing and displayed differential DNA methylation patterns in chickens that differed in disease resistance [35]. They also found that the methylation level of miRNA promoters is high [35]. However, functional study on methylation of miRNA promoters in MD is limited.

Aberrant DNA methylation is a well-known feature of cancer cells. miRNAs have been shown to function as targets in gene hypermethylation in malignant cells. Increased methylation level of tumor suppressor miRNAs in turn results in overexpression of the oncogenic targets [36–38]. Epigenetic silencing of the tumor suppressor miR-124a by aberrant DNA hypermehtylation in colorectal cancer cell line HCT-116 resulted in its target gene cyclin D kinase-6 overexpression and tumor suppressor gene retinoblastoma phosphorylation, both of which affects cell cycle progression [39]. And interestingly, miR-29 family reverted global gene methylation by targeting *DNMT3a* and *DNMT3b* directly in non-small-cell lung cancer (NSCLC) and acute myeloid leukemia cells [15, 16]. The promoter CpG islands of miR-130b was not methylated in NSCLC [40]. However, it had an increased methylation frequency in breast cancer [41]. The promoter of miR-130b~301b cluster was significantly hepermethylated in prostate cancer tissues compared with morphologically normal ones, which impaired cellular senescence and fuel malignant transformation [42]. MiR-130b hypermethylation is observed in ovarian cancer tissues and demethylation leads to reactivation of miR-130b expression in drug resistant ovarian cancer cell lines [43]. We verified that gga-miR-130b-3p was significantly downregulated in MDV-infected tumorous tissues. The upstream region of gga-miR-130b-3p gene was hypermethylated in tumorous spleens. *DNMTs* expression level increased and the catalysis of *DNMTs* contributes to its hypermethylation in tumorous spleens. *TETs* may be involved in the demethylation of 5-methylcytosine through 5-hydroxymethylcytosine as an intermediate in the chicken [44–46]. The expression of *TET2* was downregulated in tumorous spleens, which may contribute to the lower demethylation in the upstream region of gga-miR-130b-3p gene. Transcription factor binding sites that bind with the upstream region of gga-miR-130b-3p gene were predicted through online software. Some transcription factor binding sites were correlated with immune response, cell cycle regulation and tumorigenesis. Transcription factor *SP1* binding site was found in the upstream region of this miRNA. *SP1* is a zinc finger transcription factor that binds to GC-rich motifs of many gene promoters [47]. It is involved in many cellular processes, including cell growth, apoptosis, response to DNA damage and immune response [48, 49]. Many reports indicate it is overexpressed in cancers and is associated with tumorigenesis [50–53]. *SP1* plays a role in both gene transcriptional regulation and epigenetic regulation through modulating gene silencing by DNA methylation [54]. *SP1* protects the 5’ promoter regions of housekeeping and other genes from being silenced [55–57]. And it inhibits *SP1* binding when methylation spreads into *SP1* binding sites, which leads to silencing of transcription [58]. Changes in histone H3 modifications of tumor suppressor *RASSF1A* gene contribute to the eviction of *SP1* from the promoter region, and the loss of *SP1* allows CpG methylation in the promoter [59]. The *SP1* binding site was located at the CpG site 24, 25, 26, 27 in the upstream region of gga-miR-130b-3p gene and their methylation level in these four CpG sites were all higher in tumorous spleens compared with that in non-infected ones. Cyclin A-cyclin-dependent kinase (*CDK*) 2 complexes can phosphorylate *SP1*, while cyclin A-*CDK2*-mediated phosphorylation augments *SP1* DNA binding properties [60, 61]. Phosphorylation of MDV oncogene *Meq* by *CDK2* that colocalize with *Meq* drastically reduces the DNA binding activity of *Meq*, and perhaps results in the translocation of *Meq* to cytoplasm. Enhanced transport of *Meq* is profitable to the maintenance of MDV latent state. *Meq* might directly or indirectly translocate *CDK2* to coiled bodies to promote cell cycle progression during the course of transformation [62]. We assumed that MDV infection might inhibit *SP1* DNA binding properties owing to loss of *CDK2* phosphorylation. *SP1* has the function of preventing the gene promoter from methylation and thus *SP1* might be prevented from binding to the promoter of gga-miR-130b-3p, which results in the methylation of this miRNA. However, it remains to be validated through further study.

Aberrant methylation of the gene promoter regions in both suppressor and oncogenic miRNAs is involved in all key processes related to tumor development of neoplastic phenotype: uncontrolled cell proliferation, bypassed apoptotic program, and ability for invasion and metastasis spread [9, 63]. We analyzed the effects of gga-miR-130b-3p on tumorous cell features. Its downregulation in MDV-infected tissues implicates its involvement in MD tumorigenesis. Gga-miR-130b-3p expression level was slightly lower in MDV-infected CEF cells [64], which was similar with our data. Overexpression of gga-miR-130b-3p remarkably inhibited MSB1 cell proliferation at different time points. In GH3 rat pituitary tumor cells, miR-130b inhibited cell proliferation by arresting the cells in the G1 and G2 phase of the cell cycle through targeting cyclin A2 gene [65]. MiR-130b arrested cell cycle at S phase and induced apoptosis in human pancreatic cancer PANC-2 and ASPC-1 cell lines, which resulted in suppression in cell proliferation both *in vitro* and *in vivo* [66].

MiR-130b inhibited cell migration and invasion in epithelial ovarian cancer cells [67]. Overexpression of miR-130b remarkably inhibited the invasive ability of pancreatic cancer cells [66]. Moreover, a number of miRNA genes susceptible to methylation are closely correlated with cell migration, metastasis, and adhesion [9]. The overexpression of gga-miR-130b-3p prohibited the migration ability of MSB1 cells. Matrix metalloproteinases (*MMPs*) are known to be involved in tumor invasion and metastasis [68–70]. *MMP2* and *MMP9* are two important matrix proteinases in the *MMP* family that degrade type IV collagen, a major component of the basement membrane in cancers [71]. Gga-miR-130b-3p inhibited the expression level of *MMP2* and *MMP9*, which indicates that this miRNA may be involved in MD tumor transformation through suppressing cell invasion.

The hypermethylation may contribute to the downregulation of gga-miR-130b-3p in MDV-infected tumorous tissues. Overexpression of gga-miR-130b-3p inhibited cell proliferation, migration and invasion, indicating that this miRNA may function as a tumor suppressor to be involved in MD tumor transformation.

## MATERIALS AND METHODS

### Ethics statements

All animal experiment procedures and sample collection strictly followed the protocols approved by the Animal Care and Use Committee of China Agricultural University (Approval ID: XXCB-20090209), and this study was carried out in strict accordance with the guidelines and regulations established by this committee and has been approved by review board of our university.

### Sample collection

MDV-infected tumorous spleens and livers were obtained from a previously reported experiment [18]. All tissues were stored at −80°C in RNA fixer. Total RNA was isolated from these tissues to detect differential expression of miRNAs.

### Bisulfite treatment

Genomic DNA from the spleens was extracted by traditional phenol and chloroform methods. The quality of DNA was evaluated by 0.8% agarose gel electrophoresis using 100 ng extracted DNA. The concentration was detected by NanoDrop (GE Healthcare, USA) spectrophotometer, and then a total of 1.5 μg genomic DNA from each sample was treated with sodium bisulfite using an EZ DNA Methylation-Gold Kit (Zymo Research, Orange, CA) according to the manufacturer's protocol.

### Quantitative methylation analysis

The upstream 1 kb region of gga-miR-130b-3p gene was determined using Ensembl Genome Browser (http://asia.ensembl.org) and CpG islands of this region were predicted using Methprimer (http://www.urogene.org/methprimer/). PCR primers were designed by using Methyl Primer Express Software of ABI company (Applied Biosystems, Foster City, CA). Two amplicons were designed to cover the upstream region of precursor gga-miR-130b-3p. Converted DNA was amplified with two sequential PCR using the following nested primers: PCR1:5’-GTGATTAGTTAAGGGATAAAGT-3’ and 5’-AAACAACTCACCCAATTCTC-3’, PCR2:5’-TGA TTAGTTAAGGGATAAAGTTAGGT-3’ and 5’-CATC TTCCATTTTCACCCACT-3’. For each PCR, 25uL pre-mix, 1 μM of forward and reverse primers were used in a 50 μL total reaction volume. Converted DNA (100 ng) was used as the template of the first PCR. The product (5 μL) from 1^st^ PCR was used as the template for the second PCR. The first PCR amplification was performed as follows: 95°C for 3 min, 50 cycles of 95°C for 5s, 60°C for 20s, 72°C for 20s, and 72°C for 10 min, finally incubation at 4°C. The second PCR amplification was performed as follows: 95°C for 5 min, 25 cycles of 95°C for 10s, 60°C for 25s, 72°C for 45s, and a final extension of 72°C for 10 min. The 2^nd^ PCR products were purified by gel extraction from a 1% agarose gel, sub-cloned into a pGEM T-Easy vector (Promega, Madison, WI, USA) and then transformed into *Escherichia coli* (strain DH5α) using standard procedures. Methylation state of each CpG site was analyzed by randomly sequencing 10 clones.

### Cell culture and miRNA transfection

The MDV-transformed lymphoid cell line MDCC-MSB1, which was kindly provided by Division of Avian Infectious Diseases, Harbin Veterinary Research Institute of Chinese Academy of Agricultural Sciences in May, 2017, was cultured in RPMI-1640 (Invitrogen, Carlsbad, CA) with 10% fetal bovine serum (Invitrogen). The cell line was maintained in a sterile incubator at 37°C, 95% humidity, and 5% CO2. The cells were authenticated by testing ten chicken microsatellite sites (MCW0295, MCW0103, ADL0176, MCW0183, MCW0081, MCW0256, ADL0267, MCW0078, MCW0034, and ADL0210) when we first obtained the cells and started the experiment. FuGENE HD (Promega) was used to transfect cells according to manufacturer's instructions. The gga-miR-130b-3p agomir and corresponding NC were purchased from GenePharma Company (GenePharma Co. Ltd., Shanghai, China).

### Cell proliferation and migration assays

Cell proliferation was detected using the Cell Counting Kit-8 (CCK-8) (Beyotime, Shanghai, China). The cells were seeded into 96-well plates (3×10^4^ cells/well) and transfected with gga-miR-130b-3p agomir or agomir NC. Cell proliferation was detected at 24, 36, 48, 60 and 72 h post transfection. The absorbance at 450 nm was measured using a microplate spectrophotometer. All the experiments were performed independently in six replicates.

For migration assay, it was performed using the transwell. In total of 1.5×10^5^ cells were seeded into 24-well plates and transfected with gga-miR-130b-3p agomir or agomir NC. After 48 h post transfection, cells were collected and washed with PBS. Firstly, 5×10^4^ cells in serum-free medium were placed into the upper chamber of an insert (8-mm pore size; BD Bioscience, San Jose, CA). Medium containing 20% fetal bovine serum was added to the lower chamber. After 16-18 h of incubation, the cells remaining on the upper membrane were removed, and the cells that had migrated through the membrane were counted by a microscope. Each assay was performed in two replicates.

### RNA isolation and real-time PCR analysis

Total RNA was isolated from frozen samples or MSB1 cells using TRIzol Reagent (Invitrogen) according to the manufacturer's protocol. Total RNA was reverse transcribed by cDNA synthesis kit (miRACLE, USA) for miRNA expression detection. The qRT-PCR for miRNA was conducted by qPCR miRNA kit (miRACLE) according to manufacturer's protocol. Specific forward primer for gga-miR-130b-3p was 5’-AGTGCAATAATGAAAGGGCGTAA-3’. Specific forward primer for internal control 5S was 5’-ACCGGGTGCTGTAGGCTTAA-3’. Each individual sample for gga-miR-130b-3p detection was run in triplicate. The optimum thermal cycling parameters were 95°C for 10 min, 40 cycles of 95°C for 10 s, 57°C for 20 s, and 72°C for 1 min.

Total RNA was reverse transcribed by EasyScript First-Strand cDNA Synthesis SuperMix (TransGen, Beijng, China) for specific gene detection. Real-time PCR reactions for specific genes were performed with the Power SYBR Green PCR Master Mix (Invitrogen) in the ABI 7500 System (Applied Biosystems) in triplicate. Primers for *MMP2 and MMP9* genes used for the RT-qPCR were are shown in Table 1. Primers for *DNMT1, DNMT3a and DNMT3b* genes used for the RT-qPCR were reffered from Tian et al. (2013) [23]. Primers for *TET1, TET2 and TET3* genes used for the RT-qPCR were reffered from Okuzaki et al. (2017) [46].The relative genes expression level was calculated with reference to expression of 5S and β-actin. The results are described as the fold change determined by 2^−ΔΔCt^ method. The data are expressed as means ± standard errors (SEs).

**Table 1:** Primers for genes used in the RT-qPCR

Gene	Direction	Sequence
β-actin	Forward	5’-GAGAAATTGTGCGTGACATCA-3’
	Reverse	5’-CCTGAACCTCTCATTGCCA-3’
MMP2	Forward	5’-TGAAACAGGAGATTTGGAT-3’
	Reverse	5’-CATTTTGGCTTTCTTGGA-3’
MMP9	Forward	5’-ACCTGGACCGTGCCGTGAT-3’
	Reverse	5’-TGCCTCGCCGCTGTAAAT-3’

### Enzyme-linked immunosorbent assay (ELISA)

The levels of the protein products of *MMP2* and *MMP9* genes were detected with ELISAs (BG, Shanghai, China). Cell supernatants were collected and centrifuged at 1,000 × *g* for 15 min to remove debris. Cells were suspended in phosphate-buffered saline and subjected to ultrasonication. The ELISAs were conducted according to the manufacturer's protocol.

### Statistical analysis

The data are expressed as means ± SEs. All analyses were analyzed by a Student's *t*-test using the SAS system (SAS Institute Inc., Cary, NC). Values were considered to be significantly different when *P* < 0.05.

## SUPPLEMENTARY MATERIALS FIGURES AND TABLES





## Abbreviations

MD: Marek's disease
MDV: Marek's disease virus
miRNA: microRNA
d.p.i: days post infection
DNMT: DNA methyltransferase
CCK-8: Cell Counting Kit-8
SE: standard error
SP1: stimulating protein 1
NC: negative control
NSCLC: non-small-cell lung cancer
CDK: cyclin-dependent kinase
MMP: matrix metalloproteinase
TET: Tet ten-eleven translocation protein
ELISA: enzyme-linked immunosorbent assay
