# Supplementary material for: Gga-miR-130b-3p inhibits MSB1 cell proliferation, migration, invasion, and its downregulation in MD tumor is attributed to hypermethylation
Source: Oncotarget. 2018 May 11;9(36):24187–98. doi: 10.18632/oncotarget.24679 (PMC5966247; doi:10.18632/oncotarget.24679)
Supplement: Supplementary file 1 [file oncotarget-09-24187-s001.pdf]

Gga-miR-130b-3p inhibits MSB1 cell proliferation, migration, invasion, and its downregulation in MD tumor is attributed to hypermethylation

SUPPLEMENTARY MATERIALS

Supplementary Table 1: Transcription factor binding sites of upstream region of gga-miR-130b-3p gene

See Supplementary File 1
